# Oil adsorption ability of three-dimensional epicuticular wax coverages in plants

**DOI:** 10.1038/srep45483

**Published:** 2017-04-03

**Authors:** Elena V. Gorb, Philipp Hofmann, Alexander E. Filippov, Stanislav N. Gorb

**Affiliations:** 1 Department of Functional Morphology and Biomechanics, Zoological Institute, Kiel University, Am Botanischen Garten 9, Kiel, 24098, Germany; 2 Department N5, Donetsk Institute for Physics and Engineering, R. Luxemburg Str. 72, Donetsk 83112, Ukraine

## Abstract

Primary aerial surfaces of terrestrial plants are very often covered with three-dimensional epicuticular waxes. Such wax coverages play an important role in insect-plant interactions. Wax blooms have been experimentally shown in numerous previous studies to be impeding locomotion and reducing attachment of insects. Among the mechanisms responsible for these effects, a possible adsorption of insect adhesive fluid by highly porous wax coverage has been proposed (adsorption hypothesis). Recently, a great decrease in insect attachment force on artificial adsorbing materials was revealed in a few studies. However, adsorption ability of plant wax blooms was still not tested. Using a cryo scanning electron microscopy approach and high-speed video recordings of fluid drops behavior, followed by numerical analysis of experimental data, we show here that the three-dimensional epicuticular wax coverage in the waxy zone of *Nepenthes alata* pitcher adsorbs oil: we detected changes in the base, height, and volume of the oil drops. The wax layer thickness, differing in samples with untreated two-layered wax coverage and treated one-layered wax, did not significantly affect the drop behavior. These results provide strong evidence that three-dimensional plant wax coverages due to their adsorption capability are in general anti-adhesive for insects, which rely on wet adhesion.

Epicuticular waxes are cuticular lipids, which are complex mixture of cyclic (e. g. triterpenoids) and long-chain aliphatic compounds, such as primary and secondary alcohols, primary aldehydes, fatty acids, and alkanes^1^,^2^, deposited onto the plants surface^3^. They cover all aerial primary surfaces of higher plants in the form of relatively smooth two-dimensional films or layers, from which also three-dimensional projections can emerge^4^. Two-dimensional waxes vary in thickness from extremely thin films consisting of a few molecular layers in aquatic plants to noticeable crusts up to 0.5 mm thick in land plants. Three-dimensional wax projections, which originate by self-assembly eg refs 5, 6, 7, 8, 9, range in size from 0.5 to 100 μm^1^,^10^ and show various morphologies ─ platelets, rodlets, tubules, threads etc.^1^. The morphology of wax projections depends on the chemical composition of the wax and is determined by the dominating chemical compound or compound class^1^,^2^,^11^.

Epicutucular waxes play an important role in interactions between plants and their environment, also in insect-plant interactions. They protect plants against herbivory, are involved in the so called greasy pole syndrome preventing the robbery of nectar and other resources by ants, serve as a selective barrier protecting associated ants against non-specialised ant species on stems of myrmecophylic species, impair attachment, locomotion, and foraging behaviour of predatory and parasitoid insects, contribute to temporary capture of pollinators in plants with kettle trap flowers, and play a key role in prey capturing and retention by trapping organs (pitchers) of carnivorous plants (reviewed by Gorb and Gorb^12^).

It is a well-known fact that plant surfaces bearing wax projections decrease the insect locomotion (reviews by Eigenbrode^13^ and Müller^14^). The effect of the three-dimensional wax coverages on insect attachment has been experimentally studied using different approaches with a number of insect species and some plant species. Insects usually showed successful attachment to the smooth surfaces without wax or with removed wax bloom, but performed poorly on the surfaces covered with wax projections (see review by Gorb and Gorb^12^). Not only the presence of wax, but also the projection size and density of the wax coverage may affect insect attachment. This was observed on plant surfaces and additionally revealed on bio-inspired artificial wax-covered surfaces^15^,^16^. However, most plant wax surfaces studied only temporarily reduced the attachment ability of insects^17^.

We have previously proposed four hypotheses explaining the mechanisms of insect adhesion reduction on plant surfaces covered with three-dimensional epicuticular waxes^17^. (1) Wax projections create micro-roughness, which greatly decreases the real contact area between the plant surface and insect attachment organs called adhesive pads (roughness hypothesis). (2) Wax projections are easily detachable structures contaminating insect pads (contamination hypothesis). (3) Highly porous wax coverage may absorb the fluid secretion from the insect pad surface (fluid absorption hypothesis). (4) Insect pad secretion may dissolve epicuticular plant waxes (wax dissolving hypothesis). This would result in the appearance of a thick layer of fluid, making the plant surface slippery.

Recently, only the first two hypotheses have been experimentally tested. The effect of surface roughness on insect attachment has been revealed in a number of studies showing the worst insect attachment on rough substrates with a nominal asperity size of 0.3 and 1.0 μm e.g. refs 18, 19, 20, 21 corresponding to those in most wax-covered plant surfaces. Also the contamination of insect pads by the plant waxes has been detected for several insect and plant species^22^,^23^,^24^,^25^,^26^,^27^. It has been found that plants differ in their contaminating effects on insect pads: the contaminating ability depended on the micro-morphology of the wax projections and was related to both the largest dimension and the aspect ratio of the projections^27^.

As for the fluid adsorption hypothesis, only a very few relevant studies demonstrating the effect of the fluid reduction on the insect attachment to artificial surfaces have been performed. The essential reduction or even the loss of the attachment ability on smooth substrates after treating the pads of the bug *Rhodnius prolixus* with lipid solvent or after walking of the aphid *Aphis fabae* on silica gel for a certain time were recorded^28^,^29^. A significant attachment force reduction caused by a decrease of the pad secretion was measured in the stick insect *Carausius morosus* on smooth polyimide substrates that selectively adsorbed the watery component of the secretion^30^. Also rough nano-porous substrates highly reduced the attachment forces of the beetle *Coccinella septempunctata* if compared to smooth solid substrates^31^. Since the comparison of the changes in contact angles of water and oil between solid and nano-porous surfaces indicated a strong adsorption ability of the nano-porous samples for both polar and non-polar fluids, it was concluded that due to their high porosity, the nano-porous samples can adsorb the fluid from insect pads, thereby reducing insect attachment.

However, information on the adsorption ability of three-dimensional epicuticular waxes in plants is lacking in literature. The aim of the present study was to test whether the wax coverage of the waxy zone in the pitcher of the carnivorous plant *Nepenthes alata* is able to adsorb fluids. The wax bloom of *N. alata* was used here as a model plant system because it has been comprehensively studied previously in regards to its micro-morphology^26^,^32^,^33^,^34^ as well as physicochemical^35^ and material properties^26^,^36^. The main functions of this pitcher surface are prey trapping and retention fulfilled through the reduction of insect attachment e.g. refs 25,26,37 and 38. Additionally, the two-layered structure of the wax coverage (Fig. 1) made it possible to vary the porous layer thickness.

We performed experiments with the *N. alata* pitcher surfaces bearing either intact, two-layered wax coverage (UL) or only one, lower wax layer (LL) after the upper wax projections were mechanically removed. As in nature, the upper wax projections can be dislodged from the waxy zone by insects trying to escape from the trap, we used both UL and LL pitcher samples in this study. The artificial smooth solid sample (SS) served as a reference surface. We measured adsorption rates of two fluids – polar double-distilled water and non-polar mineral oil. Water was used to simulate the watery phase of the bi-phasic (water-oil emulsion) adhesive pad secretion reported for locusts, stick insect, ants, and flies e.g. refs 18,39, 40, 41, 42, 43, 44, 45. The oil was employed as a rough approximation of the oily pad secretion found previously in beetles e.g. refs 46, 47, 48, 49, 50.

## Results

### Modification of the water drops

With the only exception of the contact angles (94.50 ± 1.09° on SS, 140.76 ± 3.67° on UL, 151.60 ± 2.84° on LL) (Fig. 2a), the behavior of the water drops was very similar on all tested surfaces. We observed no changes in the drop shape during the experimental time of 30 s (see Supplementary Movie S1). The drop base usually remained the same. The drop height either did not change or was reduced by up to 1.5% of the initial value on both UL and LL or by 0.5–5.0% of the initial value on SS. The loss of the drop volume accounted for 0.5–2% of the initial value. The volume *V* decreased gradually and showed similar linear dependence on time *t* for all tested surfaces: *V* = −0.001 *t* (n = 7) on SS, *V* = −0.0013 *t* (n = 6) on UL and *V* = −0.0008 *t* (n = 6) on LL (one way ANOVA: F_2,18_ = 3.118, P = 0.072) (Fig. 2b).

### Modification of the oil drops

In the case of the oil, which readily wetted all test surfaces (Fig. 3, upper row), the drops behaved differently during 30 s of the experiment depending on the surface (Fig. 3). On SS (Fig. 4 and Supplementary Movie S1), the spreading was accompanied by the continuous growth of the drop base (by 21–38% of the initial value) and continuous decrease in the drop height (by 25–40% of the initial value) (Fig. 3 (SS) and 4a,b). These processes occurred much faster at small times and were slower at large times. Some reduction in the drop volume (by 5–8.5% of the initial value) happened at three different regimes/stages: the initial stage 1 at small times (up to 3–6 s), some intermediate stage 2 and the stage 3 at large times (Fig. 4c). The relative loss of the volume *V*/*V*_*0*_ showed the exponential character ln(*V*/*V*_*0*_) = γ*t* with γ = −0.0020 ± 0.0025 during the “quick” stage 1, whereas it could be described well by the power-law function ln(*V*/*V*_*0*_) = αln(*t*) with α = −0.0121 ± 0.0103 during the “slow” stage 3 (Fig. 4d,e).

**Figure 1:**
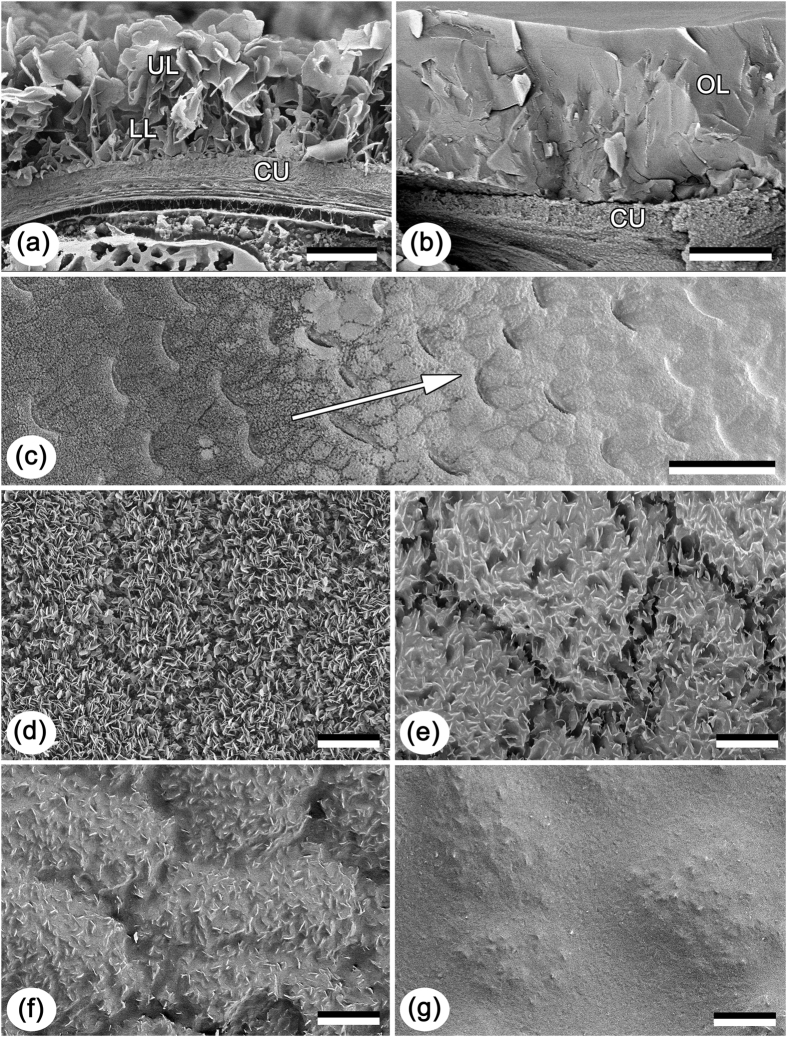
Cryo scanning electron microscopy (SEM) images of the wax coverage in the *Nepenthes alata* pitcher. (**a**) intact; (**b**–**g**) after applying the oil drops onto the pitcher surface. (**a**,**b**): cryo fracture; (**c**–**g**): view from above. Arrow in (**c**) indicates the gradient from uncovered (dark) to completely wetted/oil-adsorbing (bright) regions shown at high magnifications in (**d**–**g**), respectively. CU, cuticle; LL, lower wax layer; OL, oil adsorbed by the wax coverage; UL, upper wax layer. Scale bars: 2 μm (**a**,**b**), 10 μm (**d**–**g**) and 100 μm (**c**).

**Figure 2:**
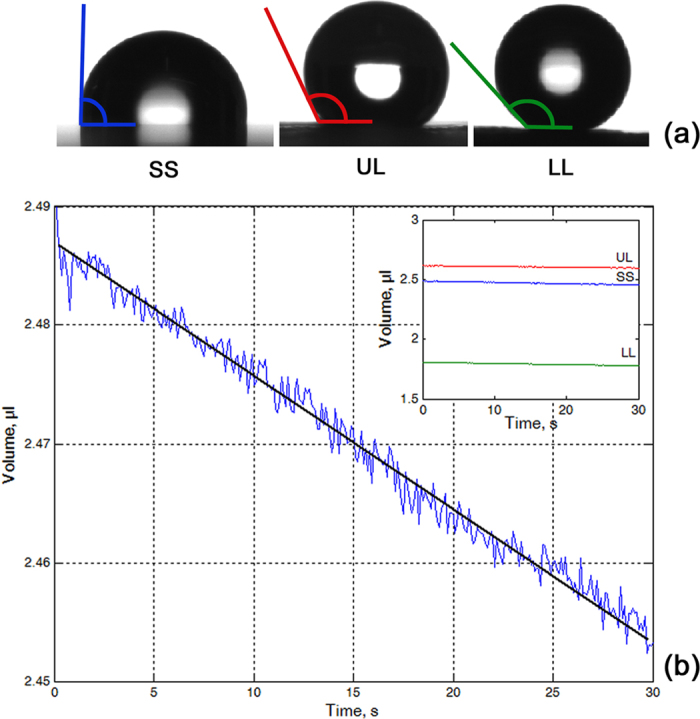
The water drops on the test surface samples. (**a**) Contact angles on the artificial smooth solid sample SS and two *Nepenthes alata* pitcher samples with two wax layers UL and one wax layer LL. (**b**) Time-dependant values of the volume of the water drop on the smooth solid sample SS. The strait black line indicates the linear fitting function *V* = −0.001 *t*. The inset shows the change of the volume on all tested surfaces: the smooth solid sample (SS, blue) and the pitcher samples with one (LL, green) and two wax layers (UL, red).

Cryo SEM investigations of the oil drops applied onto the pitcher wax coverage revealed the high affinity between oil and the wax material: fluid readily wetted and completely penetrated the wax coverage down to the cuticle (Fig. 1b–g).

**Figure 3:**
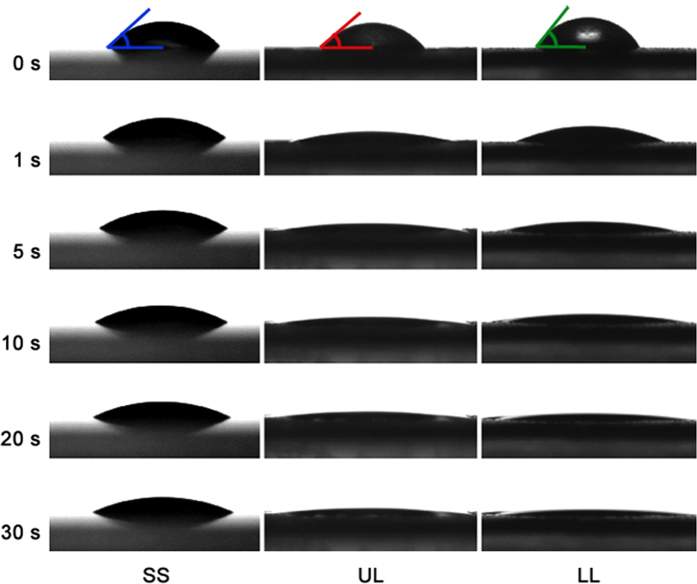
Modification of the oil drops on the test surface samples. Video sequences of the drop behavior on the smooth solid sample SS and the pitcher samples with the two-layered wax UL and one-layered wax LL during 30 s of the experiments: at 0 s, 1 s, 5 s, 10 s, 20 s and 30 s after deposition of the drops on the sample surface.

**Figure 4:**
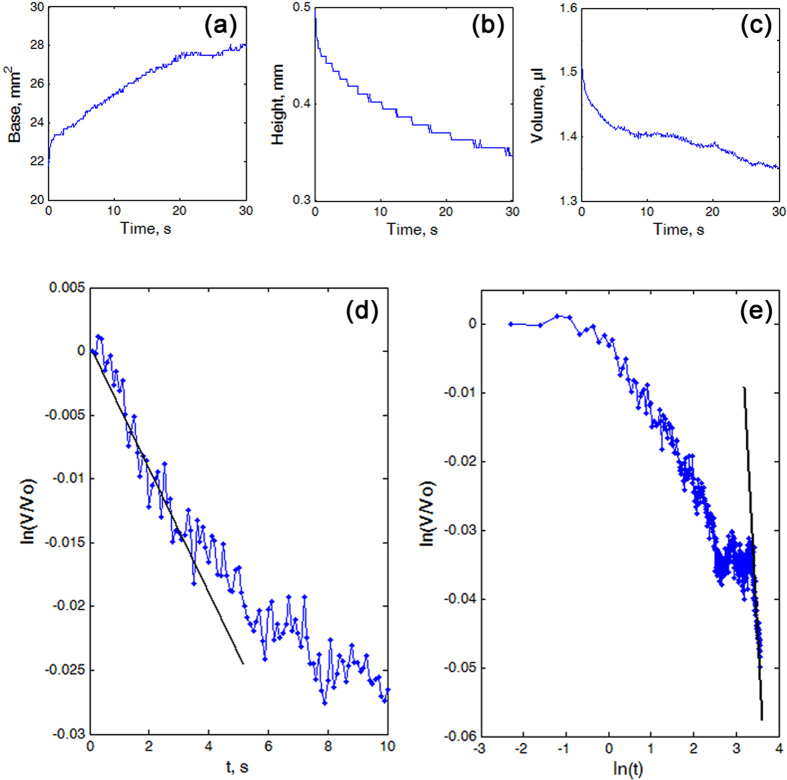
Modification of the oil drops on the smooth solid sample SS. (**a**–**c**) Time-dependant values of the base (**a**), height (**b**) and volume of the drop (**c**). (**d**,**e**) Log-linear (**d**) and log-log plots (**e**) of the time-dependence of the change in the oil drop volume *V*/*V*_*0*_. The strait black lines touching the experimental curves in (**d**) and (**e**) correspond to the exponential and scaling dependencies in different time intervals, respectively.

**Figure 5:**
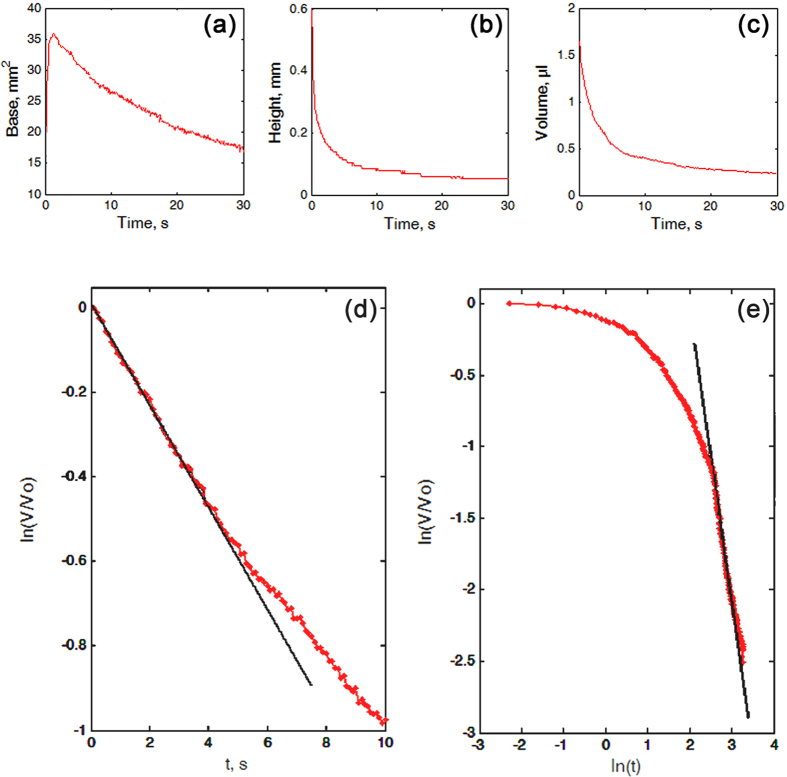
Modification of the oil drops on the two-layered wax sample of the pitcher UL. (**a**–**c**) Time-dependant values of the base (**a**), height (**b**) and volume of the drop (**c**). (**d**,**e**) Log-linear (**d**) and log-log plots (**e**) of the time-dependence of the change in the oil drop volume *V*/*V*_*0*_. The strait black lines touching the experimental curves in (**d**) and (**e**) correspond to the exponential and scaling dependencies in different time intervals, respectively.

The oil drops showed extremely quick spreading over the UL surface during the first several seconds; then after a certain time without observable changes, they sunk in either over the same drop base or with its shrinkage (Fig. 3 (UL) and Supplementary Movie S3). The intensive spread of the drop base (by 30–220% of the initial value) occurred extremely fast during the first several seconds, then the base either almost did not change or went down (by 33–57% of the highest value) (Fig. 5a). The drop height decreased by 62–88% of the initial value exceedingly quickly at the very beginning and much slower at larger times (Fig. 5b). The great reduction in the drop volume, which accounted by 40–88% of the initial value, happened through three stages: the “quick” stage 1 at small (first 2–5 s, up to10 s) times (exponential function ln(*V*/*V*_*0*_) = γ*t*, γ = −0.0783 ± 0.0513), some intermediate stage 2 and the “slow” stage 3 at large times (power-law function ln(*V*/*V*_*0*_) = αln(*t*) with α = −0.4525 ± 0.1993) (Fig. 5c–e).

**Figure 6:**
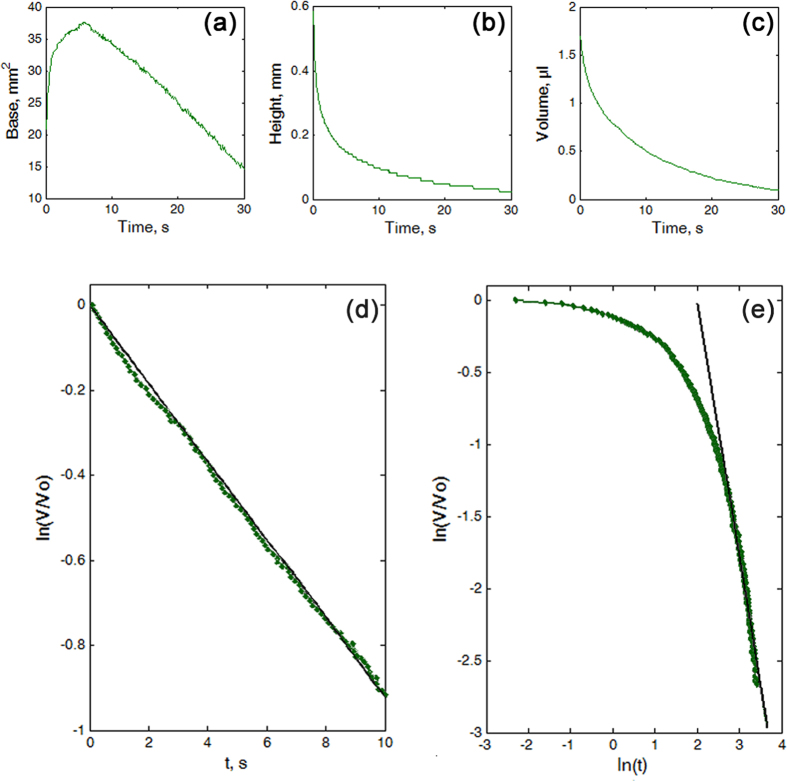
Modification of the oil drops on the one-layered wax sample of the pitcher LL. (**a**–**c**) Time-dependant values of the base (**a**), height (**b**) and volume of the drop (**c**). (**d**,**e**) Log-linear (**d**) and log-log plots (**e**) of the time-dependence of the change in the oil drop volume *V*/*V*_*0*_. The strait black lines touching the experimental curves in (**d**) and (**e**) correspond to the exponential and scaling dependencies in different time intervals, respectively.

On LL, the oil drops spread very quickly over the surface during the first several up to 10 s, then did not change in shape for a short time and afterwards started to shrink/disappear (Fig. 3 (LL) and Supplementary Movie S4). The drop base increased extremely fast during the first few (up to maximum 10) seconds by 41–160% of the initial value, then decreased either slightly (only by 10% of the maximal value) or greatly (by 40–75% of the maximal value) (Fig. 6a). The drop height decreased by 73–95% of the initial value: first (from several to up to 10 s) faster, then slower (Fig. 6b). The great decrease in the drop volume (by 32–94% of the initial value) occurred during the “quick” stage at small (0–10 s) times (exponential function ln(*V*/*V*_*0*_) = γ*t* with γ = −0.0644 ± 0.0262) and the “slow” stage at larger times (power-law function ln(*V*/*V*_*0*_) = αln(*t*) with α = −0.5100 ± 0.1790) (Fig. 6c–e). The intermediate stage 2 was either not present or very short.

**Figure 7:**
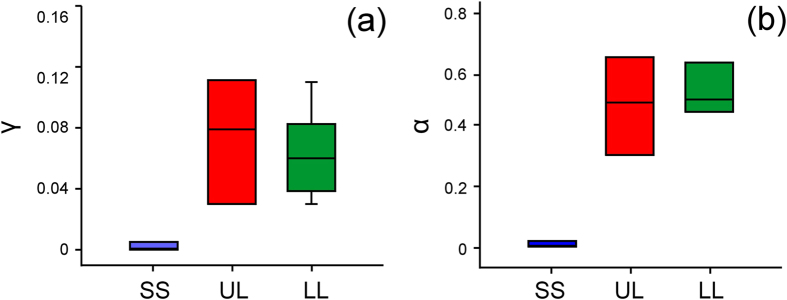
γ- and α-values obtained for the test samples. The γ- (**a**) and α- (**b**) values were calculated for fits for the exponential ln(*V*/*V*_*0*_) = γ*t* and power-law ln(*V*/*V*_*0*_) = αln(*t*) functions, respectively, obtained for the artificial smooth solid sample SS (n = 6) and pitcher samples with two wax layers UL (n = 8) and one wax layer LL (n = 9).

The comparisons of γ-values in the exponential functions (for the “quick” stage at small times) and α-values in the power-law functions (for the “slow” stage at large times) (Fig. 7) showed significant differences between surfaces (γ: H_2,22_ = 12.904, P = 0.002; α: H_2,22_ = 10.857, P = 0.002; both Kruskal-Wallis one way ANOVA on ranks). Both values were considerably lower on SS compared to both UL and LL and did not differ between the latter two surface samples (Table 1).

## Discussion

Our experiments with the water drops on the artificial smooth solid sample (SS) and two *N. alata* pitcher samples with either one (LL) or two wax layers (UL) demonstrated very similar behavior of the fluid, despite of some differences in the surface wettability between the samples. Permanence of the drop shape and base as well as very little, linear decrease in the drop height and volume during the experimental time indicate that neither spreading nor adsorption of water took place on the tested samples. Little changes in the drop height and volume resulted most likely from the evaporation of the fluid. Hence, non-significant difference in k-values (*V* = k*t*) between different samples means that similar processes, apparently evaporation of water, occur at similar rates on the tested surfaces.

We have found that performance of the oil drops differed essentially from that of the water drops. On all tested surfaces, distinct changes in shape of the oil drops as well as growth of the drop base together with the reduction of the drop height were clearly seen, especially at small times, and losses of the drop volume were revealed. We also detected certain dissimilarities in the drop behavior if compare SS with both UL and LL.

The continuous, but relatively moderate spreading of the oil drop over SS, which was relatively rapid at the beginning and slow at high times, resulted in the expanding drop base and the reducing drop height. Changes in both drop parameters were essentially lower than those in UL and LL. The obtained decrease in the oil drop volume on SS appeared to be higher than the expected one caused by the evaporation of oil. This happened possibly due to errors of experimental and/or calculation methods used in the study. However, taking into account great differences in the volume loss values between SS compared to both UL and LL, these errors can be neglected. As both γ-value (ln(*V*/*V*_*0*_) = γ*t*) and α-value (ln(*V*/*V*_*0*_) = αln(*t*)) in SS were nearly 35 times lower than those in UL and LL, we can conclude that SS did not adsorb oil, whereas both UL and LL could do this.

For the oil drops on both UL and LL, we suggest the following scenario. During the initial stage, which was usually shorter (just several seconds) in UL and longer (up to 10 s) in LL, the liquid simultaneously quickly penetrated into the porous media located under the drop and spread over the surface. This stage contributed to the most decrease of the drop volume observed in the experiments, especially on UL. At large times (starting from ca. 10^th^ s after the drop deposition on the surface), mainly imbibition of the liquid to lateral directions into the porous media, which resulted in the shrinkage of the drop base (more pronounced on LL compared to UL) and the expanding of the wetted region inside the porous layer, took place. During the intermediate stage, which was noticeable in UL, the pores under the drop base became saturated and the adsorption of the fluid towards the lateral directions into the porous media started. At that time, the drop base did not expand or even slightly decreased. The presence of this stage on UL may be explained by the higher thickness of the porous layer and, consequently, longer pores (both 2–3 times larger than those in LL^32^). We assume that the evaporation of the oil, which should occur at all stages, had probably a minimal effect on the loss of the drop volume compared to the adsorption of the fluid by the porous layer. Large values of the drop volume loss and non-significant differences in both γ- and α-values on UL and LL indicate that both one-layered and two-layered waxes of the *N. alata* pitcher were equally effective in adsorbing oil.

Our results on the behavior of the oil drops on both *N. alata* pitcher samples (with one wax layer LL and with the two-layered wax UL) are in line with the previous results on the spreading of small liquid drops of silicone oil over dry porous layers in the case of complete wetting^51^. Our observations also clearly showed that the drop motion was caused by the fluid adsorption through the porous media (see a great decrease in the drop volume in Figs 5c and 6c) resulting in the spreading of the drop over already saturated regions of the porous substrates (growth of the drop base) and the imbibition of the liquid from the drop into the porous materials (decrease of the drop base) (see Figs 5a and 6a).

Our previous study on the insect attachment to nano-porous artificial samples, showing the pronounced ability to oil adsorption, revealed extremely poor attachment of ladybird beetles *Coccinella septempunctata* to these substrates due to possible absorption of the secretion fluid from insect adhesive pads by the porous media^31^. In the case of epicuticular plant waxes, due to the high affinity between the wax material and oily insect pad secretion, the fluid disappears rapidly from the contact. That is why all kinds of forces contributing to the wet adhesion (capillary interactions and viscous adhesion) will be reduced or even completely eliminated. This will cause a great reduction in the attachment of insect to such substrates. Since the amount of the fluid secreted by insects is extremely small (average amount in the footprint is 0.986 μm^3^ in the beetle and 0.019 μm^3^ in the fly^52^) and much smaller than the adsorption capability of the waxy zone in *Nepenthes* pitcher shown in the present paper, the fluid will be immediately and completely absorbed from the insect pad by the plant wax. The results of the present study provide strong evidence that three-dimensional epicuticular wax coverages in plants are in general anti-adhesive for insects, which rely on wet adhesion.

## Methods

### Samples

Three types of surface samples were used in the study: (1) the *N. alata* pitcher sample with one wax layer of the three-dimensional epicuticular wax coverage (LL), (2) the pitcher sample with the two-layered wax (UL), and (3) the artificial smooth solid sample (SS) served as a reference surface.

Pitchers of the carnivorous plant species *N. alata* Blanco (Nepenthaceae), endemic to the Philippines^53^, were obtained from plants grown in the greenhouse at the Botanical Garden of the Kiel University (Kiel, Germany). The waxy zone of the freshly harvested pitchers was used to get both UL and LL. The untreated pitcher surface bearing both wax layers^26^,^32^,^33^,^36^ was used as UL (Fig. 1a). The LL was obtained after mechanical removal of the upper wax layer by treating the waxy zone with a two-component polyvinylsiloxane (Coltène Whaledent Dentalvertriebs GmbH, Konstanz, Germany). For this purpose, the fluid polyvinylsiloxane was applied to the pitcher surface and then peeled off after 5 min of polymerization. As SS, we used the epoxy resin sample obtained from a smooth clean glass slide by applying a two-step molding method according to Gorb^54^.

## Experiments

### Cryo scanning electron microscopy

Pieces of ca. 5 × 5 mm were cut out of the central part in the waxy zone of the *N. alata* pitcher using a razor blade. Two types of the pitcher samples were examined: (1) with the intact epicuticular wax coverage and (2) after applying a small droplet of mineral oil (light oil (neat) BioReagent M8410, Sigma-Aldrich Chemie GmbH, Munich, Germany; density 0.84 g/cm^3^ at 25 °C, viscosity 20.5 mm^2^/s at 40 °C, Brookfield viscosity 30.0 cps at 25 °C) onto the wax coverage. This oil was selected, because its viscosity corresponds to that previously estimated experimentally for the footprints of beetles^55^. The total mount specimens with the inner pitcher surface facing up and fractured samples were examined in a scanning electron microscope Hitachi S-4800 (Hitachi High-Technologies Corp., Tokyo, Japan) equipped with a Gatan ALTO 2500 cryo preparation system (Gatan Inc., Abingdon, UK). Sample preparation and mounting are described in detail by Gorb and Gorb^32^ and Benz *et al*.^33^.

### Apparent contact angle measurements

Contact angles of double-distilled water (surface tension = 72.1 mN/m, dispersion component = 19.9 mN/m, polar component = 52.2 mN/m)^56^ on test samples were measured by using a high-speed optical contact angle measuring device OCAH 200 (DataPhysics Instruments GmbH, Filderstadt, Germany) according to the sessile drop method. For a detailed description of the method, see Gorb and Gorb^35^. We applied 2 μl drops and circle or ellipse fitting for evaluation of the apparent contact angles. On each surface, the contact angles of 10 drops were measured. Data are given as mean ± SD.

### Adsorption experiments

In the adsorption experiments, we used the sessile drops (ca. 2 μl) of two fluids — polar double-distilled water and non-polar mineral oil (light oil (neat) BioReagent M8410, Sigma-Aldrich Chemie GmbH, Munich, Germany). The behavior of the drops during the first 30 s after the drop deposition on the test surfaces was video recorded (10 frames per second) using the contact angle measuring device mentioned above. On each sample type, 6 – 9 experiments were carried out with each fluid. By applying the numerical analysis of the experimental results, described below, we obtained time-dependencies for the drop base, height and volume.

### Numerical analysis of experimental data

Numerical analysis of the experimental results was organized as follows. We copied the sequence of graphic images from an experimentally recorded movie to MatLab software and transformed them into black and white maps. Numerically, these black and white maps corresponded to the matrices, which contained only 0 and 1 values. This allowed one to determine formally borders of the visualized liquid drops. As during our numerous experiments on contact angles measurements, both previous^32^,^35^ and performed in this study, we observed mostly symmetric drops, we treated here every liquid drop as cylindrically symmetric.

In a further numerical procedure, the instant height of the drop *h* was divided into a large number of as small as possible layers *dh*«1. In fact, the number of the layers was defined by the number of pixels in the recorded HD-movie. Each layer in an instant drop image had a diameter, which was determined by the borders of its projection on the vertical plane. It can be formally done in MatLab, where a black and white image corresponded to a matrix containing 0 and 1 elements only.

Using the obtained array of the diameters for all layers, one can easily calculate the areas of the circles corresponding to each layer. In particular, the lowest (first) layer of this array defined instant contact area (drop base). One could also multiply the area of every layer by the height *dh* to calculate its volume and thus collecting the volumes of all the layers up to the total volume of the drop, performing a numerical summation on all of them.

The above procedure could be easily repeated for every frame of the movie. As a result, we obtained time-dependant values of the drop volume, base and height. This procedure is illustrated in the numerically generated Supplementary Movie S5. Mutual correspondence between the real image of the process, a black and white map of the vertical drop projection (with its borders marked by the colored points), and time-dependant volume, the base and height of the drop were directly seen from a comparison between different subplots of the movie frames.

In particular, it could be seen directly from the frames that different stages of the process had different rates and different (functional) time dependence. Because of this, it was also useful to plot all time-dependant values in the log-log scale. It allowed us to separate the time intervals, where the corresponding values behaved as a linear, exponential or scaling (power-law) function of time.

Determination of different functional time-dependencies in log-linear and log-log plots (recorded for the same process, shown in the Supplementary Movie S5) is illustrated in the Supplementary Movie S6. Straight lines touching the experimental curve in the first and second subplots of this movie correspond to the exponential and scaling hypothetic functional dependencies in different time intervals, respectively.

## Additional Information

**How to cite this article:** Gorb, E. V. *et al*. Oil adsorption ability of three-dimensional epicuticular wax coverages in plants. *Sci. Rep.* **7**, 45483; doi: 10.1038/srep45483 (2017).

**Publisher's note:** Springer Nature remains neutral with regard to jurisdictional claims in published maps and institutional affiliations.

## Supplementary Material

Supplementary Movie S1

Supplementary Movie S2

Supplementary Movie S3

Supplementary Movie S4

Supplementary Movie S5

Supplementary Movie S6

Supplementary Information

## Figures and Tables

**Table 1:** Results of multiple pairwise comparisons (Dunn’s method) of γ-values and α-values obtained for the relative volume change of the oil drops on different samples.

Comparison	DF	Q	P < 0.05
γ			
SS vs UL	12.125	3.310	yes
SS vs LL	10.944	3.062	yes
UL vs LL	1.1818	0.358	no
α			
SS vs UL	9.563	2.832	yes
SS vs LL	10.500	3.031	yes
UL vs LL	0.938	0.306	no

DR, difference on ranks; LL, pitcher sample with only lower wax layer; P, probability value; SS, smooth epoxy resin replica; Q, test statistics; UL, pitcher sample with both wax layers.

## References

[b1] Barthlott W. . Classification and terminology of plant epicuticular waxes. Bot. J. Linn. Soc. 126, 237–260 (1998).

[b2] Jetter R., Kunst L. & Samuels A. L. Composition of plant epicuticular waxes in Biology of the plant cuticle (ed. Riederer M. & Müller C.) 145–181 (Blackwell, 2006).

[b3] Jeffree C. E. The fine structure of the plant cuticle in Biology of the plant cuticle (ed. Riederer M. & Müller C.) 11–125 (Blackwell, 2006).

[b4] Jeffree C. F. The cuticle, epicuticular waxes and trichomes of plants, with reference to their structure, function and evolution in Insects and the plant surface (ed. Juniper B. & Southwood R.) 23–64 (Edward Arnold Publishers, 1986).

[b5] Jeffree C. E., Baker E. A. & Holloway P. J. Ultrastructure and recrystallisation of plant epicuticular waxes. New Phytol. 75, 539–449 (1975).

[b6] Jetter R. & Riederer M. Epicuticular crystals of nanocosan-10 ol: *in vitro* reconstitution and factors influencing crystal habits. Planta 195, 257–270 (1994).

[b7] Jetter R. & Riederer M. *In vitro* reconstitution of epicuticular wax crystals: formation of tubular aggregates by long chain secondary alkanediols. Bot. Acta 108, 111–120 (1995).

[b8] Meusel I., Neinhuis C., Markstadter C. & Barthlott W. Chemical composition and recrystallization of epicuticular waxes: coiled rodlets and tubules. Plant Biol. 2, 462–470 (2000).

[b9] Koch K. & Ensikat H. J. The hydrophobic coatings of plant surfaces: epicuticular wax crystals and their morphologies, crystallinity and molecular self-assembly. Micron 39, 759–772 (2008). 18187332 10.1016/j.micron.2007.11.010

[b10] Koch K., Bhushan B. & Barthlott W. Multifunctional plant surfaces and smart materials in Handbook of nanotechnology (ed. Bhushan B.) 1399–1436 (Springer, 2010).

[b11] Bargel H., Koch K., Cerman Z. & Neinhuis C. Structure–function relationships of the plant cuticle and cuticular waxes — a smart material? Funct. Plant Biol. 33, 893–910 (2006). 10.1071/FP06139 32689300

[b12] Gorb E. V. & Gorb S. N. Anti-adhesive surfaces in plants and their biomimetic potential in Materials design inspired by nature: function through inner architecture (ed. Fratzl P., Dunlop J. W. C., Weinkamer R.) 282–309 (RSC Publishing, 2013).

[b13] Eigenbrode S. D. Plant surface waxes and insect behaviour in Plant cuticles — an integrated functional approach (ed. Kerstiens G.) 201–222 (BIOS Scientific Publishers, 1996).

[b14] Müller C. Plant-insect interactions on cuticular surfaces in Biology of the plant cuticle (ed. Riederer M. & Müller C.) 398–422 (Blackwell, 2006).

[b15] Gorb E., Voigt D., Eigenbrode S. D. & Gorb S. Attachment force of the beetle *Cryptolaemus montrouzieri* (Coleoptera, Coccinellidae) on leaf surfaces of mutants of the pea *Pisum sativum* (Fabaceae) with regular and reduced wax coverage. Arthropod-Plant Interact. 2, 247–259 (2008).

[b16] Gorb E. . Insect attachment on crystalline bioinspired wax surfaces formed by alkanes of varying chain lengths. Beilstein J. Nanotechnol. 5, 1031–1041 (2014). 25161838 10.3762/bjnano.5.116 PMC4143128

[b17] Gorb E. V. & Gorb S. N. Attachment ability of the beetle *Chrysolina fastuosa* on various plant surfaces. Entomol. Exp. Appl. 105, 13–28 (2002).

[b18] Gorb S. N. Attachment devices of insect cuticle (Kluwer Academic Publishers, 2001).

[b19] Peressadko A. & Gorb S. Surface profile and friction force generated by insects in *Proceedings of the First International Industrial Conference* Bionik. (ed. Boblan I. & Bannasch R.) 257–263 (VDI Verlag, 2004).

[b20] Voigt D., Schuppert J. M., Dattinger S. & Gorb S. N. Sexual dimorphism in the attachment ability of the Colorado potato beetle *Leptinotarsa decemlineata* (Coleoptera: Chrysomelidae) to rough substrates. J. Insect Physiol. 54, 765–776 (2008). 18387627 10.1016/j.jinsphys.2008.02.006

[b21] Bullock J. M. & Federle W. Division of labour and sex differences between fibrillar, tarsal adhesive pads in beetles: effective elastic modulus and attachment performance. J. Exp. Biol. 212, 1876–1888 (2009). 19483006 10.1242/jeb.030551

[b22] Juniper B. E. & Burras J. K. How pitcher plants trap insects. New Sci. 269, 75–77 (1962).

[b23] Stork N. E. Role of waxblooms in preventing attachment to brassicas by the mustard beetle, *Phaedon cochleariae*. Entomol. Exp. Appl. 26, 100–107 (1980).

[b24] Eigenbrode S. D., Castognola T., Roux M. B. & Steljes L. Mobility of three generalist predators is greater on cabbage with glossy leaf wax than on cabbage with a wax bloom. Entomol. Exp. Appl. 81, 335–343 (1999).

[b25] Gaume L. . How do plant waxes cause flies to slide? Experimental tests of wax-based trapping mechanisms in three pitfall carnivorous plants. Athropod Struct. Dev. 33, 103–111 (2004). 10.1016/j.asd.2003.11.005 18089026

[b26] Gorb E. . Composite structure of the crystalline epicuticular wax layer of the slippery zone in the pitchers of the carnivorous plant *Nepenthes alata* and its effect on insect attachement. J. Exp. Biol. 208, 4651–4662 (2005). 16326946 10.1242/jeb.01939

[b27] Gorb E. V. & Gorb S. N. Do plant waxes make insect attachment structures dirty? Experimental evidence for the contamination hypothesis in *Ecology and biomechanics — a mechanical approach to the ecology of animals and plants* (ed. Herrel A., Speck T. & Rowe N. P.) 147–162 (CRC Press, 2006).

[b28] Edwards J. S. & Tarkanian M. The adhesive pads of Heteroptera: a re-examination. Proc. R. Entomol. Soc. Lond. A 45, 1–5 (1970).

[b29] Dixon A. F. G., Croghan P. C. & Gowing R. P. The mechanism by which aphids adhere to smooth surfaces. J. Exp. Biol. 152, 243–253 (1990).

[b30] Dirks J.-H., Clemente C. J. & Federle W. Insect tricks: two-phasic foot pad secretion prevents slipping. J. R. Soc. Interface 7, 587–593 (2010). 19755498 10.1098/rsif.2009.0308 PMC2842779

[b31] Gorb E. V., Hosoda N., Miksch C. & Gorb S. N. Slippery pores: anti-adhesive effect of nanoporous substrates on the beetle attachment system. J. R. Soc. Interface 7, 1571–1579 (2010). 20427333 10.1098/rsif.2010.0081 PMC2988254

[b32] Gorb E. & Gorb S. Functional surfaces in the pitcher of the carnivorous plant Nepenthes alata: a cryo-SEM approach in Functional surfaces in biology: adhesion related effects (ed. Gorb S. N.) 205–238 (Springer, 2009).

[b33] Benz M. J., Gorb E. V. & Gorb S. N. Diversity of the slippery zone microstructure in pitchers of nine carnivorous *Nepenthes* taxa. Arthropod-Plant Interact. 6, 147–158 (2012).

[b34] Gorb E. V., Baum M. J. & Gorb S. N. Development and regeneration ability of the wax coverage in *Nepenthes alata* pitchers: a cryo-SEM approach. Sci. Rep. 3, 3078 (2013). 24165663 10.1038/srep03078 PMC3810656

[b35] Gorb E. V. & Gorb S. N. Physicochemical properties of functional surfaces in pitchers of the carnivorous plant *Nepenthes alata* Blanco (Nepenthaceae). Plant Biol. 8, 841–848 (2006). 17203436 10.1055/s-2006-923929

[b36] Gorb E. V., Purtov J. & Gorb S. N. Adhesion force measurements on the two wax layers of the waxy zone in *Nepenthes alata* pitchers. Sci. Rep. 4, 5154 (2014). 24889352 10.1038/srep05154 PMC4042122

[b37] Gaume L., Gorb S. & Rowe N. Function of epidermal surfaces in the trapping efficiency of *Nepenthes alata* pitchers. New Phytol. 156, 479–489 (2002). 10.1046/j.1469-8137.2002.00530.x 33873580

[b38] Scholz I. . Slippery surfaces of pitcher plants: *Nepenthes* wax crystals minimize insect attachment via microscopic surface roughness. J. Exp. Biol. 213, 115–1125 (2010). 10.1242/jeb.035618 20228348

[b39] Voetsch W. . Chemical composition of the attachment pad secretion of the locust *Locusta migratoria*. Insect Biochem. Mol. Biol. 32, 1605–1613 (2002). 12429112 10.1016/s0965-1748(02)00098-x

[b40] Drechsler P. & Federle W. Biomechanics of smooth adhesive pads in insects: influence of tarsal secretion on attachment performance. J. Comp. Physiol. A 192, 1213–1222 (2006). 10.1007/s00359-006-0150-5 16835787

[b41] Dirks J.-H. & Federle W. Fluid-based adhesion in insects - principles and challenges. Soft Matter 7, 11047–11053 (2011).

[b42] Betz O. . Peptide mass analyses of the tarsal adhesive secretion in the desert locust *Schistocerca gregaria* and the Madagascar hissing cockroach *Gromphadorhina portentosa*. Insect Mol. Biol. 25, 541–549 (2016). 27126627 10.1111/imb.12241

[b43] Gerhardt H., Betz O., Albert K. & Lämmerhofer M. Similarities, dissimilarities and classification of molecular (hydrocarbon) profiles of insect secretions in dependence of species, sex, and sampled body region. J. Chem. Ecol. 42, 725–738 (2016). 27380036 10.1007/s10886-016-0718-7

[b44] Gerhardt H., Schmitt C., Betz O., Albert K. & Lämmerhofer M. Contact solid-phase microextraction with uncoated glass and polydimethylsiloxane-coated fibers versus solvent sampling for the determination of hydrocarbons in adhesion secretions of Madagascar hissing cockroaches *Gromphadorrhina portentosa* (Blattodea) by gas chromatography-mass spectrometry. J. Chromatogr. A 1388, 24–35 (2015). 25728659 10.1016/j.chroma.2015.02.027

[b45] Reitz M. . Analysis of chemical profiles of insect adhesion secretions by gas chromatography-mass spectrometry. Anal. Chim. Acta 854, 47–60 (2015). 25479867 10.1016/j.aca.2014.10.056

[b46] Ishii S. Adhesion of a leaf feeding ladybird *Epilachna vigintioctomaculta* (Coleoptera: Coccinellidae) on a vertically smooth surface. Appl. Entomol. Zool. 22, 222–228 (1987).

[b47] Kosaki A. & Yamaoka R. Chemical composition of footprints and cuticula lipids of three species of lady beetles. Jpn. J. Appl. Entomol. Zool. 40, 47–53 (1996).

[b48] Eisner T. & Aneshansley D. J. Defense by foot adhesion in a beetle (*Hemisphaerota cyanea*). Proc. Natl. Acad. Sci. USA 97, 6568–6573 (2000). 10841556 10.1073/pnas.97.12.6568 PMC18661

[b49] Attygalle A. B., Aneshansley D. J., Meinwald J. & Eisner T. Defense by foot adhesion in a chrysomelid beetle (*Hemisphaerota cyanea*): characterization of the adhesive oil. Zoology 103, 1–6 (2000).

[b50] Geiselhardt S. F., Geiselhardt S. & Peschke K. Comparison of tarsal and cuticular chemistry in the leaf beetle *Gastrophysa viridula* (Coleoptera: Chrysomelidae) and an evaluation of solid-phase microextraction and solvent extraction techniques. Chemoecology 19, 185–193 (2009).

[b51] Starov V. M., Zhdanov S. A., Kosvintsev S. R., Sobolev V. D. & Velarde M. G. Spreading of liquid drops over porous substrates. Adv. Colloid Interface Sci. 104, 123–158 (2003). 12818493 10.1016/s0001-8686(03)00039-3

[b52] Peisker H. & Gorb S. N. Evaporation dynamics of tarsal liquid footprints in flies (*Calliphora vicina*) and beetles (*Coccinella septempunctata*). J. Exp. Biol. 215, 1266–1271 (2012). 22442363 10.1242/jeb.065722

[b53] McPherson S. Pitcher plants of the Old World (Redfern Natural History Productions, 2009).

[b54] Gorb S. N. Visualisation of native surfaces by two-step molding. Microsc. Today 15, 44–46 (2007).

[b55] Peisker H., Heepe L., Kovalev A. & Gorb S. N. Comparative study of the fluid viscosity in tarsal hairy attachment systems of flies and beetles. J. R. Soc. Interface 11, 1–7 (2014). 10.1098/rsif.2014.0752 PMC4233759 25142527

[b56] Busscher H. J., Vanpert A. W. J., Deboer P. & Arends J. The effect of the surface roughening of polymers on measured contact angle of liquids. Colloids Surf. 9, 319–331 (1984).

