# Supplementary material for: Oil adsorption ability of three-dimensional epicuticular wax coverages in plants
Source: Sci Rep. 2017 Apr 3;7:45483. doi: 10.1038/srep45483 (PMC5377368; doi:10.1038/srep45483)
Supplement: Supplementary Information [file srep45483-s7.doc]

Oil adsorption ability of three-dimensional epicuticular wax coverages in plants

**Elena V. Gorb, Philipp Hofmann, Alexander E. Filippov, and Stanislav N. Gorb**

**Supplementary Information**

**Supplementary Movie S1.** Behavior of the water drop on the two-layered wax sample of the *Nepenthes alata* pitcher UL during 30 s after deposition of the drop on the sample surface.

**Supplementary Movie S2.** Behavior of the oil drop on the smooth solid sample SS during 30 s after deposition of the drop on the sample surface.

**Supplementary Movie S3.** Behavior of the oil drop on the two-layered wax sample of the *Nepenthes alata* pitcher UL during 30 s after deposition of the drop on the sample surface.

**Supplementary Movie S4.** Behavior of the oil drop on the one-layered wax sample of the *Nepenthes alata* pitcher LL during 30 s after deposition of the drop on the sample surface.

**Supplementary Movie S5.** Numerically generated movie illustrating the obtained time-dependant values of the drop volume, base and height. Mutual correspondence between the real image of the process, black and white map of the vertical drop projection (with its borders marked by the colored points) and time-dependant values are seen from a comparison between different subplots of the movie frames. 1 s in the movie corresponds to 6 s of the real time.

**Supplementary Movie S6.** Numerically generated movie illustrating the determination of different functional time-dependencies in log-linear and log-log plots (recorded for the same process shown in the Supplementary Movie S5). Straight lines touching the experimental curves in the left and right subplots correspond to the exponential and scaling hypothetic functional dependencies in different time intervals, respectively.
